# Developing a Comprehensive Model for Explaining Treatment Adherence Behaviour: Evidence From the International Beamer Consortium

**DOI:** 10.1002/puh2.70377

**Published:** 2026-10-11

**Authors:** Francisco Lupiáñez‐Villanueva, Damien S. E. Broekharst, Eline J. Mertens, Claire Everitt, Aad R. Liefveld, Giuseppe Fico, Sjaak Bloem

**Affiliations:** ^1^ PredictBy Research and Consulting Barcelona Spain; ^2^ Department of Information and Communication Science Universitat Oberta de Catalunya Barcelona Spain; ^3^ Center for Marketing & Supply Chain Management Nyenrode Business University Breukelen the Netherlands; ^4^ Devices Centre of Excellence, Pfizer Cambridge UK; ^5^ Link2Trials Hilversum the Netherlands; ^6^ PACE Clinical Utrecht the Netherlands; ^7^ Life Supporting Technologies Research Group Universidad Politécnica de Madrid Madrid Spain; ^8^ Janssen‐Cilag B.V., Johnson & Johnson Breda the Netherlands

**Keywords:** acceptance, control, health behaviour, health consciousness, medication compliance, subjective health experience, treatment adherence behaviour, treatment concerns, treatment priority

## Abstract

**Introduction:**

Optimizing treatment adherence behaviour has become a focus in healthcare, as improved adherence enhances clinical outcomes, patient experiences and health system sustainability. For this optimization endeavour scholars have posited diverse variables divided into two clusters. The first variable cluster emphasizes treatment priority, treatment concerns and health consciousness. The second variable cluster highlights acceptance, control and subjective health experience. Although some of these variables have been tested in isolation, few comprehensive models have been established. Therefore, this study aims at establishing a comprehensive model for explaining treatment adherence behaviour.

**Method:**

An online questionnaire was administered to 4081 participants from six different countries. This questionnaire covered sample characteristics as well as instruments measuring health consciousness, treatment priority, treatment concerns, acceptance, control, subjective health experiences, medication compliance, health behaviour and treatment adherence behaviour. Data were analysed using descriptive, reliability, validity and model statistics.

**Result:**

All concepts in the established model were measured reliably and showed sufficient validity. Most associations between concepts were stable and statistically significant across the overall and segmented models, except for those associating treatment concerns with adherence behaviour and control with subjective health experience in the previous month. The analysis shows that health consciousness is modestly to moderately positively associated with treatment adherence indirectly via 12 intermediary pathways, four of which were consistently significant, two were largely nonsignificant, and the remaining pathways exhibited mixed patterns. Between 22.6% and 38.5% of variance was explained by the models.

**Conclusion:**

This model offers valuable insights into treatment adherence behaviour yet remains open to further refinement and development, either by striving for greater parsimony or by embracing relational complexity through data‐driven machine learning approaches.

## Graphical abstract

This study developed and validated a comprehensive model of treatment adherence behaviour using survey data from 4081 participants across six countries. The measurement instruments demonstrated good reliability and validity, and the model explained 22.6%–38.5% of the variance in treatment adherence behaviour with most relationships being statistically significant.

## 1 Introduction

In recent decades, an increased emphasis has been placed on the optimization of treatment adherence in healthcare as better treatment adherence behaviour among patients seems to reliably and consistently enhance clinical outcomes, patient experiences as well as health system sustainability and resilience [1, 2, 3]. The concept of treatment adherence behaviour is defined by the World Health Organization as the extent to which the behaviour of an individual corresponds with the mutually established recommendations and guidance provided by a healthcare professional [4]. In pursuit of optimizing treatment adherence behaviour among patients, an interdisciplinary coalition of scholars has devoted considerable effort to crafting sophisticated explanations of this particular concept, yielding a diverse and wide‐ranging array of both hypothetical and empirically substantiated variables [5]. This emerging, dynamic and still evolving corpus of contemporary scientific literature generally delineates these proposed variables into two distinct principal clusters [6, 7, 8, 9, 10, 11, 12, 13, 14, 15].

The first cluster of variables stems from a body of research focussed on health awareness, which frequently highlights three essential concepts: treatment priority, treatment concerns and health consciousness [6, 7, 8, 9, 10, 11]. Treatment priority denotes the extent to which an individual perceives an intervention as indispensable for the effective management or resolution of their health condition [6, 7]. Treatment concerns encapsulate the range of challenges that an individual associates with different aspects of a required medical intervention, including side effects, potential risks or burden of treatment [6, 7]. Health consciousness denotes the extent of an individual's awareness and attentiveness regarding their own health, coupled with a proactive disposition to adopt health‐promoting behaviours and take preventive actions [8, 9]. Additionally, research shows that the relationship between these variables and treatment adherence behaviour may be mediated by medication compliance, which denotes the extent to which an individual follows medication instructions [10, 11].

The second cluster of variables emerged from a corpus of scientific literature focussed on health perception, which mostly emphasizes three crucial concepts: acceptance, control and subjective health experience [12, 13, 14]. Acceptance expresses the extent to which individuals are able to experience their own health condition as an integral part of their existence [12, 13, 14]. Control expresses the extent to which individuals believe themselves to be able to exert influence over their own health condition [12, 13, 14]. Subjective health experience refers to an individual's experience of physical and mental functioning while living his or her life the way he or she wants to, within the actual constraints and limitations of individual existence [12, 13, 14]. Additionally, research shows that the relationship between these variables and treatment adherence behaviour may be mediated by health behaviour, which denotes the lifestyle behaviours, actions, habits and choices an individual exhibits to influence their health [15].

Although the relationships between some of the aforementioned variables and treatment adherence behaviour have been theoretically considered and empirically tested in existing frameworks (e.g., necessity‐concern framework [6, 7], health belief model [16, 17] and subjective health experience model [12, 13, 14]), these attempts were isolated, sporadic and fragmented in nature [5]. Given the paucity of coherent efforts levied at configuring these variables within a theoretically substantiated framework, the present study seeks to redress this gap by systematically interrelating them in a comprehensive model designed to elucidate their direct and indirect relationships as well as explain treatment adherence behaviour (see Figure 1). Additionally, this treatment adherence behaviour model is also segmented along the lines of important sample characteristics (i.e., age, gender, environment, education, employment, habitation and country) in order to explore the consistency of associations in the model and provide more insight into treatment adherence behaviour of subgroups. Such a comprehensive treatment adherence behaviour model could facilitate healthcare professionals in comprehending how, what and when care and support should be delivered in order to optimize treatment adherence behaviour among their patients.

**FIGURE 1:**
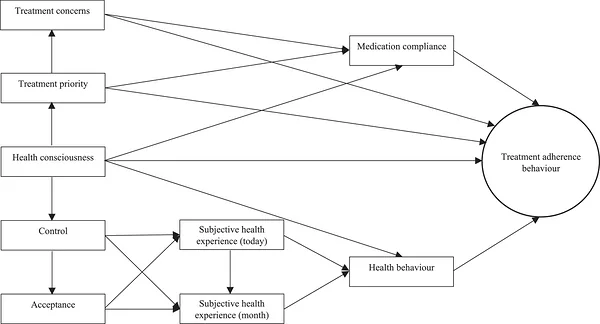
Treatment adherence behaviour model.

## 2 Methods

### 2.1 Design, Procedure and Participants

This study was undertaken within the context of the international BEAMER consortium, which is a major European initiative focussed on developing a comprehensive behavioural model to comprehend and ameliorate treatment adherence behaviour across a range of diseases [18]. In order to examine the aforementioned treatment adherence behaviour model, a quantitative research approach was employed, utilizing online questionnaires. These surveys were disseminated between January and April 2024 by the SAGO and Link2trails research agency to a considerable group of participants from six countries: the United States of America, Germany, France, Spain, Norway and the Netherlands. Eligibility for participation required individuals to be at least 18 years old with a minimum of one chronic disease. Participants were invited via email, accompanied by detailed information about the study and a formal consent request. Only individuals who provided explicit consent for their responses to be used in future research were included in the study. No study‐specific quotas were applied as the research agencies continuously monitor the representativeness of their panels, and the study targeted a broad population rather than a specific subgroup. The research agencies configured all questionnaire items as mandatory and provided only fully completed questionnaires minimizing non‐response. The questionnaires covered a wide range of topics, beginning with sample characteristics, followed by various validated measurement tools addressing areas such as health consciousness, treatment priority, treatment concerns, acceptance, control, subjective health experiences, medication compliance, health behaviour and treatment adherence behaviour.

### 2.2 Questionnaire, Items and Scales

Sample characteristics, including age, gender, environment, education, employment, habitation and country, were captured through individual items. These items were measured using nominal scales with dichotomous response options or ordinal scales featuring ascending response categories. Treatment priority and treatment concerns were measured using their respective scales in the Beliefs about Medicine Questionnaire (BMQ) [19, 20]. Each concept was measured using five items equipped with a 5‐point Likert scale ranging from 1 = fully disagree to 5 = fully agree [19, 20]. Health consciousness was measured by utilizing the relevant scale included in the HealthStyles survey [21]. This concept was measured using five items equipped with a 5‐point Likert scale ranging from 1 = fully disagree to 5 = fully agree [21]. Medication compliance was measured by deploying the 5‐item Medication Adherence Report Scale (MARS‐5) [22]. Responses for this scale were recorded on a 6‐point Likert scale ranging from 1 = fully disagree to 6 = fully agree [22]. Acceptance and control were measured using their respective scales in the Bloem and Stalpers questionnaire [12, 13, 14]. Each concept was measured using three items equipped with a 7‐point Likert scale ranging from 1 = fully disagree to 7 = fully agree [12, 13, 14]. Subjective health experience of today and previous month was assessed using the Bloem and Stalpers ladders [12, 13, 14]. Scores were determined on an 11‐level scale in which level 1 represented the worst day and level 11 represented the best day of the previous month [12, 13, 14]. Health behaviour was appraised using an instrument on the basis of the BRAVO dimensions covering ‘exercise’, ‘nutrition’, ‘rest’, ‘smoking’, ‘alcohol use’ and ‘general health’ [15, 23, 24, 25, 26]. Responses for these dimensions were recorded on a 6‐point Likert scale ranging from 1 = fully disagree to 6 = fully agree [15, 23, 24, 25, 26]. Treatment adherence behaviour was assessed using the Perceived Behaviour Scale from the Treatment Adherence Perception Questionnaire (TAPQ) [27]. The total score was calculated by summing the responses to all six items [27]. An overview of the variables and instruments deployed in this study is provided in Table 1.

**TABLE 1:** Variables and instruments.

Variables	Instruments	Properties
Treatment concerns	Relevant scales in the Beliefs about Medicine Questionnaire (BMQ) [19, 20]	Five items with 5‐point Likert scales from 1 = fully disagree to 5 = fully agree
Treatment priority		Five items with 5‐point Likert scales from 1 = fully disagree to 5 = fully agree
Health consciousness	Relevant scale in the HealthStyles Survey [21]	Five items with a 5‐point Likert scale from 1 = fully disagree to 5 = fully agree
Control	Relevant scales in the Bloem and Stalpers Questionnaire [12, 13, 14]	Three items with a 7‐point Likert scale from 1 = fully disagree to 7 = fully agree
Acceptance		Three items with a 7‐point Likert scale from 1 = fully disagree to 7 = fully agree
Health behaviour	BRAVO Scale [15, 23, 24, 25, 26]	Five items with a 6‐point Likert scale from 1 = fully disagree to 6 = fully agree
Medication compliance	Medication Adherence Report Scale (MARS‐5) [22]	Five items with a 6‐point Likert scale from 1 = fully disagree to 6 = fully agree
Subjective health experience (previous month)	Bloem and Stalpers Ladders [12, 13]	Ladder with level 1 = worst day and level 11 = best day reflecting monthly average
Subjective health experience (today)		Ladder with level 1 = worst day and level 11 = best day reflecting level of today
Treatment adherence behaviour	Relevant scale in the Treatment Adherence Perception Questionnaire (TAPQ) [27]	Six items with a scale from 1 to 6–8 generating a summed score

### 2.3 Analysis, Interpretation and Software

Sample characteristics were defined using descriptive statistics, with continuous variables reported as means and categorical variables as percentages. Questionnaire characteristics were analysed through metrics of reliability, construct validity, convergent validity and discriminant validity. The reliability of an instrument reflects the internal consistency of its items in measuring the same underlying construct [28]. Reliability was assessed using Cronbach's alpha (*α*), rho_*a* and rho_*c* coefficients, with values classified as poor (<0.60), acceptable (0.60–0.70) or good (>0.70) [28]. The construct validity of an instrument denotes the accuracy with which it operationalizes and quantifies the underlying construct it is designed to measure [28]. Construct validity was examined through factor analysis, which confirmed and refined the factorial structure of the measurement instruments by identifying items with cross‐loadings [28]. The convergent validity of an instrument reflects the empirical correspondence between the instrument and other measures [28]. Convergent validity was evaluated through the average variance extracted (AVE), which is deemed sufficient if exceeding the 0.50 threshold [28]. The discriminant validity of an instrument denotes the empirical divergence between the instrument and other measures [28]. Discriminant validity was tested using the heterotrait–monotrait (HTMT) ratio, which was considered acceptable if below the 0.90 threshold [28]. Model characteristics were examined through regression analysis, with results presented as association sizes, significance levels and explained variance. Association sizes, represented by standardized beta coefficients (*β*), were classified as small (<0.25), medium (0.25–0.50) or large (>0.50) [28]. Significance levels, based on two‐tailed *p* values, were considered statistically significant if below the 0.05 threshold [28]. Explained variance, measured by *R*‐squared (*R* ^2^) values, was categorized as weak (<0.25), moderate (0.25–0.50) or strong (>0.50) [28]. Sample characteristics were analysed using IBM SPSS Statistics Version 27, whereas questionnaire and model characteristics were delineated using SmartPLS Version 4.0 [28, 29, 30].

## 3 Results

### 3.1 Sample Characteristics

The final sample of this study consisted of 4081 participants of which 746 participants were from the USA, 611 participants were from France, 598 participants were from Germany, 618 participants were from Spain, 917 participants were from the Netherlands, and 591 participants were from Norway. The detailed sample characteristics are presented in Table 2.

**TABLE 2:** Sample description.

	The United States	France	Germany	Spain	The Netherlands	Norway	Total
**Age**							
<60 years	462 (61.9%)	445 (72.8%)	459 (76.8%)	553 (89.5%)	647 (70.6%)	538 (91.0%)	3104 (76.1%)
≥60 years	284 (38.1%)	166 (27.2%)	139 (23.2%)	65 (10.5%)	270 (29.4%)	53 (9.0%)	977 (23.9%)
**Gender**							
Male	308 (41.3%)	263 (43.0%)	300 (50.2%)	299 (48.4%)	347 (37.8%)	342 (57.9%)	1859 (45.6%)
Female	435 (58.3%)	345 (56.5%)	297 (49.7%)	317 (51.3%)	564 (61.5%)	249 (42.1%)	2207 (54.1%)
Other	3 (0.4%)	3 (0.5%)	1 (0.1%)	2 (0.3%)	6 (0.7%)	0 (0.0%)	15 (0.3%)
**Environment**							
Urban	498 (66.8%)	273 (44.7%)	363 (60.7%)	540 (87.4%)	545 (59.4%)	358 (60.6%)	2577 (63.1%)
Rural	248 (33.2%)	338 (55.3%)	235 (39.3%)	78 (12.6%)	372 (40.6%)	233 (39.4%)	1504 (36.9%)
**Education**							
<Secondary	145 (19.4%)	131 (21.4%)	76 (12.7%)	52 (8.4%)	77 (8.4%)	114 (19.3%)	595 (14.6%)
≥Secondary	601 (80.6%)	480 (78.6%)	522 (87.3%)	566 (91.6%)	840 (91.6%)	477 (80.7%)	3486 (85.4%)
**Employment**							
Employed	300 (40.2%)	356 (58.3%)	372 (62.2%)	438 (70.9%)	427 (46.6%)	384 (65.0%)	2277 (55.8%)
Unemployed	294 (39.4%)	149 (24.4%)	158 (26.4%)	126 (20.4%)	306 (33.4%)	145 (24.5%)	1178 (28.9%)
Others	152 (20.4%)	106 (17.3%)	68 (11.4%)	54 (8.7%)	184 (20.0%)	62 (10.5%)	626 (15.3%)
**Habitation**							
Alone	192 (25.7%)	133 (21.8%)	171 (28.6%)	54 (8.7%)	234 (25.5%)	120 (20.3%)	904 (22.2%)
Together	526 (70.5%)	473 (77.4%)	421 (70.4%)	555 (89.8%)	668 (72.8%)	467 (79.0%)	3110 (76.2%)
Others	28 (3.8%)	5 (0.8%)	6 (1.0%)	9 (1.5%)	15 (1.7%)	4 (0.7%)	67 (1.6%)
**Multimorbidity**							
1 disease	424 (56.8%)	384 (62.8%)	390 (65.2%)	367 (59.4%)	557 (60.7%)	391 (66.2%)	2513 (61.6%)
2 diseases	188 (25.2%)	157 (25.7%)	135 (22.6%)	182 (29.4%)	226 (24.7%)	143 (24.2%)	1031 (25.2%)
≥3 diseases	134 (18.0%)	70 (11.5%)	73 (12.2%)	69 (11.2%)	134 (14.6%)	57 (9.6%)	537 (13.2%)
**Disease**							
Arrhythmia	24 (3.2%)	19 (3.1%)	55 (9.2%)	31 (5.0%)	68 (7.4%)	28 (4.7%)	225 (5.5%)
Arterial hypertension	47 (6.3%)	166 (27.2%)	42 (7.0%)	119 (19.3%)	25 (2.7%)	39 (6.6%)	438 (10.7%)
Asthma	124 (16.6%)	135 (22.1%)	133 (22.2%)	135 (21.8%)	202 (22.0%)	144 (24.4%)	837 (20.5%)
Bipolar disorder	44 (5.9%)	19 (3.1%)	14 (2.3%)	13 (2.1%)	28 (3.1%)	42 (7.1%)	160 (3.9%)
Cancer	40 (5.4%)	43 (7.0%)	26 (4.3%)	17 (2.8%)	40 (4.4%)	10 (1.7%)	176 (4.3%)
Chronic heart failure	10 (1.3%)	20 (3.3%)	18 (3.0%)	10 (1.6%)	26 (2.8%)	21 (3.6%)	105 (2.6%)
Chronic migraine	88 (11.8%)	83 (13.6%)	69 (11.5%)	136 (22.0%)	68 (7.4%)	76 (12.9%)	520 (12.7%)
Pulmonary disease	51 (6.8%)	14 (2.3%)	40 (6.7%)	15 (2.4%)	53 (5.8%)	16 (2.7%)	189 (4.6%)
Chronic pain syndrome	91 (12.2%)	17 (2.8%)	64 (10.7%)	23 (3.7%)	76 (8.3%)	45 (7.6%)	316 (7.7%)
Liver cirrhosis	8 (1.1%)	3 (0.5%)	5 (0.8%)	4 (0.6%)	5 (0.5%)	11 (1.9%)	36 (0.9%)
Congenital anomalies	5 (0.7%)	1 (0.2%)	5 (0.8%)	1 (0.2%)	21 (2.3%)	5 (0.8%)	38 (0.9%)
Diabetes mellitus	131 (17.6%)	94 (15.4%)	109 (18.2%)	64 (10.4%)	144 (15.7%)	66 (11.2%)	608 (14.9%)
HIV/AIDS	11 (1.5%)	7 (1.1%)	2 (0.3%)	9 (1.5%)	4 (0.4%)	2 (0.3%)	35 (0.9%)
Hypertensive heart disease	49 (6.6%)	9 (1.5%)	4 (0.7%)	11 (1.8%)	24 (2.6%)	15 (2.5%)	112 (2.7%)
IBD	34 (4.6%)	25 (4.1%)	22 (3.7%)	34 (5.5%)	34 (3.7%)	22 (3.7%)	171 (4.2%)
Ischaemic heart disease	6 (0.8%)	3 (0.5%)	2 (0.3%)	6 (1.0%)	3 (0.3%)	6 (1.0%)	26 (0.6%)
Kidney diseases	27 (3.6%)	10 (1.6%)	16 (2.7%)	16 (2.6%)	20 (2.2%)	12 (2.0%)	101 (2.5%)
Lower respiratory infection	8 (1.1%)	12 (2.0%)	14 (2.3%)	11 (1.8%)	22 (2.4%)	12 (2.0%)	79 (1.9%)
Lupus	13 (1.7%)	4 (0.7%)	2 (0.3%)	8 (1.3%)	3 (0.3%)	6 (1.0%)	36 (0.9%)
Malaria	6 (0.8%)	0 (0.0%)	1 (0.2%)	0 (0.0%)	1 (0.1%)	2 (0.3%)	10 (0.2%)
Multiple sclerosis	13 (1.7%)	11 (1.8%)	10 (1.7%)	6 (1.0%)	20 (2.2%)	16 (2.7%)	76 (1.9%)
Osteoporosis	52 (7.0%)	23 (3.8%)	20 (3.3%)	20 (3.2%)	39 (4.3%)	22 (3.7%)	176 (4.3%)
Parkinson's disease	3 (0.4%)	0 (0.0%)	3 (0.5%)	0 (0.0%)	3 (0.3%)	2 (0.3%)	11 (0.3%)
Psoriasis	46 (6.2%)	34 (5.6%)	23 (3.8%)	52 (8.4%)	56 (6.1%)	45 (7.6%)	256 (6.3%)
Rheumatic heart disease	1 (0.1%)	0 (0.0%)	2 (0.3%)	2 (0.3%)	3 (0.3%)	4 (0.7%)	12 (0.3%)
Rheumatoid arthritis	79 (10.6%)	43 (7.0%)	56 (9.4%)	43 (7.0%)	100 (10.9%)	32 (5.4%)	353 (8.6%)
Schizophrenia	7 (0.9%)	2 (0.3%)	8 (1.3%)	3 (0.5%)	10 (1.1%)	9 (1.5%)	39 (1.0%)
Stroke	21 (2.8%)	11 (1.8%)	27 (4.5%)	5 (0.8%)	22 (2.4%)	13 (2.2%)	99 (2.4%)
Tuberculosis	0 (0.0%)	3 (0.5%)	0 (0.0%)	1 (0.2%)	3 (0.3%)	5 (0.8%)	12 (0.3%)
Others	238 (31.9%)	124 (20.3%)	120 (20.1%)	167 (27.0%)	346 (37.7%)	132 (22.3%)	1127 (27.6%)

In line with other studies, participants above 60 years of age are considered older adults [31]. The subgroups with regard to other genders (e.g., non‐binary), other types of habitation (e.g., nursing homes) and specific disease categories (e.g., Parkinson's disease) will be left out of the analysis as these groups are often too small to provide reliable and valid results.

### 3.2 Instrument Characteristics

The reliability of the instrument on health behaviour (*α* = 0.67; rho_*a* = 0.67; rho_*c* = 0.80) can be considered acceptable, whereas the reliability of the instruments on health consciousness (*α* = 0.78; rho_*a* = 0.79; rho_*c* = 0.85), treatment priority (*α* = 0.85; rho_*a* = 0.86; rho_*c* = 0.90), treatment concerns (*α* = 0.81; rho_*a* = 0.82; rho_*c* = 0.87), medication compliance (*α* = 0.87; rho_*a* = 0.88; rho_*c* = 0.91), acceptance (*α* = 0.83; rho_*a* = 0.85; rho_*c* = 0.90), control (*α* = 0.82; rho_*a* = 0.84; rho_*c* = 0.89) and adherence treatment behaviour (*α* = 0.89; rho_*a* = 0.90; rho_*c* = 0.92) can be deemed good. The instruments on health consciousness, treatment priority, treatment concerns, medication compliance, acceptance, control and adherence treatment behaviour had sufficient construct validity as all their items loaded on a single component, whereas the instrument on health behaviour also had sufficient construct validity after excluding two double‐loading items (i.e., ‘alcohol use’ and ‘smoking’). The instruments on health consciousness (AVE = 0.53), treatment priority (AVE = 0.63), treatment concerns (AVE = 0.57), medication compliance (AVE = 0.66), acceptance (AVE = 0.75), control (AVE = 0.74), health behaviour (AVE = 0.50) and adherence treatment behaviour (AVE = 0.65) demonstrated sufficient convergent validity with coefficients of 0.50 or higher. The instruments on health consciousness (HTMT = 0.10–0.72), treatment priority (HTMT = 0.09–0.32), treatment concerns (HTMT = 0.10–0.32), medication compliance (HTMT = 0.05–0.50), acceptance, control (HTMT = 0.06–0.79), subjective health experience of today (HTMT = 0.09–0.56), subjective health experience of previous month (HTMT = 0.06–0.56), health behaviour (HTMT = 0.12–0.72) and adherence treatment behaviour (HTMT = 0.10–0.50) demonstrated sufficient discriminant validity as coefficients remained below the 0.90 threshold. For the instruments on subjective health experience, no reliability or convergent validity could be determined as they are comprised of a single value and are not multidimensional in nature.

### 3.3 Model Characteristics

In order to further clarify the characteristics of the comprehensive treatment adherence behaviour model, this section details the direct relationships among the model variables, the indirect pathways associating health consciousness with treatment adherence behaviour as well as the explained variance of the overall and segmented models.

#### 3.3.1 Direct Relationships

From the analysis (see Table 3 and Supporting Information 1) stems that health consciousness has a modest to moderate significant positive association with treatment priority (*β* = 0.101–0.289). Health consciousness further has a modest significant positive association with medication compliance (*β* = 0.030–0.157) except for older individuals, individuals with other types of employment and those from France, Germany and the Netherlands. Health consciousness also has a modest significant positive association with treatment adherence behaviour (*β* = 0.070–0.167) except for individuals from Spain and the Netherlands. Health consciousness subsequently has a moderate to large significant positive association with health behaviour (*β* = 0.372–0.524) and control (*β* = 0.275–0.493). Treatment priority has a modest to moderate significant positive association with treatment concerns (*β* = 0.141–0.366) and medication compliance (*β* = 0.131–0.292), whereas its minor positive association with treatment adherence (*β* = 0.063–0.221) is also significant except for older individuals and those from the USA and Germany. Treatment concerns have a modest to moderate significant negative association with medication compliance (*β* = −0.065 to −0.449) except for individuals with other types of employment, whereas their negative association with adherence behaviour (*β* = −0.006 to −0.081) is minor and often nonsignificant. Medication compliance has a moderate significant positive association with treatment adherence behaviour (*β* = 0.276–0.444). Control has a large significant positive association with acceptance (*β* = 0.642–0.746) and a modest to moderate significant positive association with subjective health experience of today (*β* = 0.110–0.325) expect for those from Germany and Norway, whereas its association with subjective health experience of previous month (*β* = 0.009–0.152) is smaller and often nonsignificant. Acceptance has a moderate significant positive association with subjective health experience of today (*β* = 0.235–0.391) and a smaller significant positive impact on subjective health experience of previous month (*β* = 0.035–0.271) except for individuals from Norway. Subjective health experience of today has a moderate to large significant positive association with subjective health experience of previous month (*β* = 0.378–0.551) and a modest to moderate significant positive association with health behaviour (*β* = 0.049–0.247) except for lower educated individuals and those from Norway. Subjective health experience of previous month also has a modest significant positive association with health behaviour (*β* = 0.047–0.244) except for individuals from France. Health behaviour has a modest to moderate significant positive association with treatment adherence behaviour (*β* = 0.141–0.307).

**TABLE 3:** Model characteristics.

	Overall (*β*)
Direct relationship	
Acceptance → subjective health experience (day)	0.306^*^
Acceptance → subjective health experience (month)	0.195^*^
Medication compliance → treatment adherence	0.402^*^
Treatment concerns → treatment adherence	−0.043^**^
Treatment concerns → medication compliance	−0.253^*^
Control → acceptance	0.672^*^
Control → subjective health experience (today)	0.176^*^
Control → subjective health experience (month)	0.064^*^
Health behaviour → treatment adherence	0.231^*^
Health consciousness → treatment adherence	0.106^*^
Health consciousness → medication compliance	0.085^*^
Health consciousness → control	0.401^*^
Health consciousness → health behaviour	0.471^*^
Health consciousness → treatment priority	0.216^*^
Treatment priority → treatment adherence	0.133^*^
Treatment priority → medication compliance	0.191^*^
Treatment priority → treatment concerns	0.263^*^
Subjective health experience (today) → health behaviour	0.164^*^
Subjective health experience (today) → subjective health experience (month)	0.454^*^
Subjective health experience (month) → health behaviour	0.166^*^
**Indirect relationship**	
Health consciousness → treatment adherence	0.191^*^
Health consciousness → health behaviour → treatment adherence	0.109^*^
Health consciousness → control → subjective health experience (today) → subjective health experience (month) → health behaviour → treatment adherence	0.001^*^
Health consciousness → control → subjective health experience (month) → health behaviour → treatment adherence	0.001^**^
Health consciousness → control → acceptance → subjective health experience (today) → subjective health experience (month) → health behaviour → treatment adherence	0.001^*^
Health consciousness → medication compliance → treatment adherence	0.034^*^
Health consciousness → control → acceptance → subjective health experience (month) → health behaviour → treatment adherence	0.002^*^
Health consciousness → treatment priority → treatment adherence	0.029^*^
Health consciousness → treatment priority → treatment concerns → medication compliance → treatment adherence	−0.006^*^
Health consciousness → control → subjective health experience (today) → health behaviour → treatment adherence	0.003^*^
Health consciousness → treatment priority → medication compliance → treatment adherence	0.017^*^
Health consciousness → treatment priority → treatment concerns → treatment adherence	−0.002^**^
Health consciousness → control → acceptance → subjective health experience (today) → health behaviour → treatment adherence	0.003^*^
**Explained variance**	**31.8%**

^**^ *p* < 0.05.

^*^ *p* < 0.001.

#### 3.3.2 Indirect Relationships

From the analysis (see Table 3 and Supporting Information 1), it stems that health consciousness showed a modest to moderate significant positive indirect association with treatment adherence behaviour (*β* = 0.154–0.247). This overall indirect relationship encapsulates the cumulative association of all intermediary pathways through which health consciousness is connected to treatment adherence behaviour. One such pathway involved health behaviour through which health consciousness shows a modest significant positive association with treatment adherence behaviour (*β* = 0.072–0.147). A subsequent pathways incorporated treatment priority and medication compliance via which health consciousness demonstrates a modest and predominantly significant positive association with treatment adherence behaviour (*β* = 0.006–0.024). A third pathway encompassed treatment priority, treatment concern and medication compliance by which health consciousness exhibits a modest and predominantly significant negative association with treatment adherence behaviour (*β* = −0.001 to −0.017). A fourth pathway included control, acceptance, subjective health experience of today, subjective health experience of previous month and health behaviour through which health consciousness reveals a modest and predominantly significant positive association with treatment adherence behaviour (*β* = 0.000–0.003). A fifth pathway comprised medication compliance via which health consciousness indicates a modest and often significant positive association with treatment adherence behaviour (*β* = 0.011–0.062). A sixth pathway contained treatment priority by which health consciousness presents a modest and often significant positive association with treatment adherence behaviour (*β* = 0.011–0.051). A seventh pathway involved control, subjective health experience of today and health behaviour through which health consciousness expresses a modest and often significant positive association with treatment adherence behaviour (*β* = 0.000–0.009). An eight pathway incorporated control, acceptance, subjective health experience of today and health behaviour via which health consciousness reflects a modest and often significant positive association with treatment adherence behaviour (*β* = 0.001–0.006). A ninth pathway encompassed control, subjective health experience of today, subjective health experience of previous month and health behaviour by which health consciousness implies a modest and often significant positive association with treatment adherence behaviour (*β* = 0.000–0.004). A tenth pathway included control, acceptance, subjective health experience of previous month and health behaviour through which health consciousness displays a modest and often significant positive association with treatment adherence behaviour (*β* = 0.000–0.003). An eleventh pathway comprised treatment priority and treatment concern via which health consciousness portrays a modest and mostly nonsignificant negative association with treatment adherence behaviour (*β* = −0.000 to −0.006). A twelfth pathway contained control, subjective health experience of previous month and health behaviour by which health consciousness establishes a modest and mostly nonsignificant positive association with treatment adherence behaviour (*β* = 0.000–0.002).

#### 3.3.3 Explained Variance

From the analysis (see Table 3 and Supporting Information 1) stems that all models explain between 22.6% and 38.5% of variance in treatment adherence behaviour with the overall model accounting for 31.8%. When stratified by age, this model explains 31.9% of variance in treatment adherence behaviour among individuals below 60 years and 22.6% among those above 60 years. When segmented by gender, this model explains 34.0% of variance in treatment adherence behaviour among men and 30.4% among women. When stratified by residential environment, this model explains 32.0% of variance in treatment adherence behaviour among urban individuals and 31.8% among rural individuals. When segmented by education level, this model explains 26.6% of variance in treatment adherence behaviour among lower educated individuals and 32.8% among higher educated individuals. When stratified by habitation status, this model explains 27.3% of variance in treatment adherence behaviour among individuals living alone and 32.9% among individuals living together. When segmented by employment status, this model explains 33.1% of variance in treatment adherence behaviour among employed individuals and 30.7% among both unemployed individuals and those in other employment situations. When stratified by country, this model explains 29.5% of variance in treatment adherence behaviour for Spain, 30.2% for the United States, 30.4% for France, 34.7% for Germany, 35.1% for the Netherlands and 38.5% for Norway.

## 4 Discussion

In this study a comprehensive model for explaining treatment adherence behaviour was established, measured and validated. Additionally, the treatment adherence behaviour model was segmented along the lines of important sample characteristics, namely, age, gender, environment, education, employment, habitation and country, in order to provide healthcare professionals and other important stakeholders with deeper insight into the treatment adherence behaviour of particular subgroups. Analysis of both the overall and segmented models yielded several important and remarkable observations regarding model associations, model variables and explained variance.

All concepts in the treatment adherence behaviour model were measured with adequate reliability and validity. However, some concepts may have a narrow definitional scope, whereas others may exhibit overlap. One example of a narrowly defined concept is treatment adherence behaviour, which focuses exclusively on adherence to medical and therapeutic treatments while neglecting adherence to broader lifestyle guidelines. In contrast, overlap may exist between the two concepts related to subjective health experience as they are rather similar in content and measurement. Notably, it should be mentioned that health consciousness emerges as a central element in the model as it demonstrates significant associations with all other concepts. Most associations between concepts were stable and statistically significant across the overall and segmented models. However, a few associations displayed less stability across the segmented models. The first association that showed instability was the small and sometimes nonsignificant negative association between treatment concerns and treatment adherence behaviour reflecting the paradoxical tendency of some individuals to become nonadherent due to uncertainty about treatment design, goals and outcomes, whereas many still turn to healthcare professionals for guidance [32]. The second unstable association was the small and sometimes nonsignificant positive association between control and subjective health experience of the previous month, which may reflect the substantial conceptual overlap between subjective health experience of today and of previous month reducing the unique contribution of control to the latter [33]. All models showed a consistently substantial proportion of explained variance in treatment adherence behaviour ranging from 22.6% to 38.5% with the overall model explaining 31.8% of the variance. However, some models exhibit markedly divergent levels of explained variance in treatment adherence behaviour. In particular, the model regarding older individuals shows lower explained variance in treatment adherence behaviour, which may be attributable to greater multimorbidity, increased treatment complexity, reduced digital access and limited health literacy in this population [34].

Notwithstanding the overall promising results yielded by this study, the model under investigation remains rather propaedeutic in nature leaving ample scope for continued refinement and development. Such further refinement and development of the model might be realized through a deliberate pursuit of greater simplicity entailing the construction of a more elegant and parsimonious model that captures the essential dynamics while eschewing superfluous complexity [35, 36]. In striving for a more parsimonious model consideration may be given to the exclusion of the constructs of treatment concerns or even treatment priority as these demonstrated rather modest associations and inconsistent levels of statistical significance with treatment adherence behaviour across both the overall and segmented models. Furthermore, it may be prudent to consider the retention of only one of the constructs relating to subjective health experience in order to enhance model parsimony given that their substantial conceptual and measurement overlap increases the likelihood of strong covariance without commensurate gains in explanatory utility. Moreover, it may be prudent to consider the consolidation of the health behaviour and treatment adherence behaviour constructs in order to enhance model parsimony given that treatment adherence behaviour principally denotes compliance with prescribed medical and therapeutic regimens, whereas health behaviour encompasses adherence to more comprehensive and expansive lifestyle recommendations. Such further refinement and development of the model might also be achieved by explicitly accommodating the complexity and nonlinearity of the relationships among its variables leveraging machine‐learning techniques that are oriented toward data‐driven pattern discovery rather than theory‐driven explanation [37, 38]. From this perspective, parsimony should not be pursued at the expense of explanatory richness warranting the inclusion of all core psychological constructs within the model. In the interest of analytical rigour and conceptual completeness, the deliberate incorporation of demographic and socioeconomic variables within these types of models may be considered both justified and necessary to ensure a comprehensive understanding of the factors influencing treatment adherence behaviour.

### 4.1 Strengths and Limitations

This study demonstrates several strengths and limitations. A key strength lies in the substantial sample size, which enhances the generalizability and representativeness of the findings. Additionally, the high reliability and validity of the measurement instruments used further strengthen the accuracy and credibility of the results. Another notable strength is the inclusion of a multi‐country sample, which improves the interpretability and applicability of the findings across diverse populations. A potential limitation is the bias of the final sample toward Western countries limiting the broader generalizability of the results. Another potential limitation is that the sample exhibits a slight skew in certain sample characteristics (e.g., female‐to‐male ratio) raising concerns about potential distortions and misrepresentation in the findings. A subsequent potential limitation relates to the self‐reported, cross‐sectional and online nature of the data collection increasing the risk of common method bias, social desirability bias, recall bias and selection bias. A further limitation is the absence of potentially important control variables (e.g., health literacy, digital literacy, healthcare accessibility, intervention availability, treatment duration and medication burden) restricting the sample characterization, subgroup analyses and statistical adjustment required for a more nuanced interpretation of the findings.

### 4.2 Practical Implications

The treatment adherence behaviour model shows that health consciousness in particular impacts treatment adherence behaviour implying that support and interventions may focus on improving the health consciousness of patients through, for instance, health education and self‐management training. The treatment adherence behaviour model also suggests that subjective health experience influences treatment adherence behaviour implying that support and interventions may emphasize enhancing subjective health experience of patients through, for example, shared decision‐making, personalized counselling and patient‐centred care delivery. The treatment adherence behaviour model further indicates that effects vary across populations implying that specific contextual factors should be considered in the design of support and intervention.

### 4.3 Future Research

Multiple directions for future inquiry merit consideration to further strengthen the explanations of treatment adherence behaviour. One avenue for future research lies in refining the treatment adherence behaviour model through the removal of redundant constructs and the merging of related variables increasing the model's parsimony and conceptual clarity. Another avenue for future research involves the application of alternative methodologies, such as machine learning algorithms, to further develop and explore explanatory models of treatment adherence based on the core model variables validated in this study. A last avenue for future research entails the qualitative explication of these explanatory models with the aim of developing a practical framework as well as associated guidelines and instruments to support healthcare professionals in improving treatment adherence.

## 5 Conclusion

This study introduced a novel model for understanding treatment adherence behaviour, which was segmented on the basis of key sample characteristics, including age, gender, environment, education, employment, habitation and country. Although the constructs inherent in the model demonstrated robust reliability, satisfactory construct, convergent and discriminant validity as well as generally strong inter‐construct relationships, the model nevertheless retains potential for further refinement and development. Further refinement and development of the model could be achieved either by pursuing greater parsimony through a more elegant specification that captures essential dynamics or by embracing relational complexity and nonlinearity via machine learning approaches oriented toward data‐driven pattern discovery. Such refinement and development could result in a model that even better enables healthcare professionals to discern how, what and when care and support ought to be delivered in order to optimize treatment adherence behaviour.

## Author Contributions

Damien S. E. Broekharst and Sjaak Bloem analysed the data and wrote the manuscript. Sjaak Bloem, Claire Everitt, Aad R. Liefveld and Francisco Lupiáñez‐Villanueva were involved in data collection. Sjaak Bloem, Eline J. Mertens, Claire Everitt, Aad R. Liefveld, Giuseppe Fico and Francisco Lupiáñez‐Villanueva reviewed and edited the manuscript. All authors have read and approved the manuscript.

## Funding

This research was funded by the Innovative Health Initiative (Grant number 101034369).

## Disclosure

The funder had no role in the design and conduct of the study; collection, management, analysis, and interpretation of the data; preparation, review, or approval of the manuscript; and decision to submit the manuscript for publication.

## Ethics Statement

This study has been approved by the Ethics Committee of the Universitat Oberta de Catalunya (CE23‐PR20). All methods were carried out in accordance with relevant guidelines and regulations.

## Consent

Written informed consent was obtained from all participants.

## Conflicts of Interest

F.L.V. was employed at PredictBy Research and Consulting. C.E. was employed at Pfizer. A.R.L. was employed at Link2trials and PACE Clinical. S.B. was employed at Janssen‐Cilag. The remaining authors declare no conflicts of interest.

## Supporting information




**Supplementary File 1**: puh270377‐sup‐0001‐SuppMat.docx

## Data Availability

The dataset used during this study is not publicly available but is available from the corresponding author upon reasonable request.
