# Supplementary material for: Developing a Comprehensive Model for Explaining Treatment Adherence Behaviour: Evidence From the International Beamer Consortium
Source: Public Health Chall. 2026 Oct 11;5(4):e70377. doi: 10.1002/puh2.70377 (PMC13654311; doi:10.1002/puh2.70377)
Supplement: Supplementary file 1 [file PUH2-5-e70377-s001.docx]

**Supplementary Material 1.** Extended model characteristics

|  | **Age (β)** | | **Gender (β)** | | **Environment (β)** | | **Education (β)** | | **Habitation (β)** | | **Employment (β)** | | | **Country (β)** | | | | | | **Overall (β)** |
| --- | --- | --- | --- | --- | --- | --- | --- | --- | --- | --- | --- | --- | --- | --- | --- | --- | --- | --- | --- | --- |
| **Direct relationship** | <60 | >60 | Male | Female | Urban | Rural | <Secondary | >Secondary | Alone | Together | Employed | Unemployed | Other | USA | France | Germany | Spain | Netherlands | Norway |  |
| Acceptance 🡪 subjective health experience (day) | 0.301* | 0.287* | 0.335* | 0.276* | 0.299* | 0.319* | 0.277* | 0.312* | 0.314* | 0.303* | 0.300* | 0.317* | 0.283* | 0.235* | 0.391* | 0.306* | 0.354* | 0.311* | 0.291* | 0.306* |
| Acceptance 🡪 subjective health experience (month) | 0.195* | 0.191* | 0.156* | 0.220* | 0.190* | 0.201* | 0.224* | 0.192* | 0.159* | 0.201* | 0.218* | 0.148* | 0.198* | 0.173* | 0.200* | 0.223* | 0.200* | 0.271* | 0.035 | 0.195* |
| Medication compliance 🡪 treatment adherence | 0.410* | 0.276* | 0.401* | 0.398* | 0.406* | 0.388* | 0.378* | 0.405* | 0.363* | 0.407* | 0.408* | 0.402* | 0.329* | 0.326* | 0.406* | 0.430* | 0.323* | 0.424* | 0.444* | 0.402* |
| Treatment concerns 🡪 treatment adherence | -0.035** | -0.014 | -0.081* | -0.018 | -0.060* | -0.007 | -0.046 | -0.042** | -0.014 | -0.049** | -0.042** | -0.024 | -0.046 | -0.064 | -0.006 | -0.050 | -0.016 | -0.060 | -0.038 | -0.043** |
| Treatment concerns 🡪 medication compliance | -0.254* | -0.124* | -0.336* | -0.182* | -0.276* | -0.203* | -0.291* | -0.247* | -0.200* | -0.267* | -0.278* | -0.263* | -0.065 | -0.312* | -0.252* | -0.234* | -0.126** | -0.115** | -0.449* | -0.253* |
| Control 🡪 acceptance | 0.673* | 0.661* | 0.664* | 0.675* | 0.688* | 0.644* | 0.716* | 0.663* | 0.679* | 0.672* | 0.663* | 0.660* | 0.697* | 0.655* | 0.679* | 0.691* | 0.719* | 0.642* | 0.746* | 0.672* |
| Control 🡪 subjective health experience (today) | 0.159* | 0.239* | 0.126* | 0.216* | 0.162* | 0.200* | 0.167** | 0.178* | 0.164* | 0.178* | 0.152* | 0.148* | 0.309* | 0.325* | 0.136** | 0.110 | 0.138** | 0.205* | 0.119 | 0.176* |
| Control 🡪 subjective health experience (month) | 0.068* | 0.040 | 0.055** | 0.077** | 0.078* | 0.042 | 0.034 | 0.069* | 0.074** | 0.062** | 0.080* | 0.047 | 0.009 | 0.071 | 0.028 | 0.028 | 0.096** | 0.046 | 0.152** | 0.064* |
| Health behaviour 🡪 treatment adherence | 0.224* | 0.257* | 0.223* | 0.238* | 0.230* | 0.239* | 0.163* | 0.242* | 0.237* | 0.226* | 0.220* | 0.231* | 0.286* | 0.244* | 0.141** | 0.214* | 0.307* | 0.230* | 0.233* | 0.231* |
| Health consciousness 🡪 treatment adherence | 0.105* | 0.116** | 0.119* | 0.096* | 0.112* | 0.099* | 0.119** | 0.104* | 0.113** | 0.105* | 0.109* | 0.108* | 0.108** | 0.142** | 0.131** | 0.160* | 0.086 | 0.070 | 0.167* | 0.106* |
| Health consciousness 🡪 medication compliance | 0.097* | 0.042 | 0.096* | 0.079* | 0.102* | 0.065** | 0.117** | 0.080* | 0.074** | 0.089* | 0.118* | 0.084** | 0.048 | 0.151* | 0.064 | 0.030 | 0.157* | 0.026 | 0.139* | 0.085* |
| Health consciousness 🡪 control | 0.374* | 0.474* | 0.389* | 0.417* | 0.414* | 0.377* | 0.421* | 0.396* | 0.427* | 0.391* | 0.354* | 0.404* | 0.493* | 0.478* | 0.452* | 0.318* | 0.398* | 0.425* | 0.275* | 0.401* |
| Health consciousness 🡪 health behaviour | 0.476* | 0.465* | 0.456* | 0.485* | 0.476* | 0.459* | 0.485* | 0.468* | 0.465* | 0.473* | 0.487* | 0.447* | 0.463* | 0.498* | 0.510* | 0.524* | 0.480* | 0.489* | 0.372* | 0.471* |
| Health consciousness 🡪 treatment priority | 0.212* | 0.221* | 0.278* | 0.173* | 0.245* | 0.169* | 0.253* | 0.211* | 0.101** | 0.256* | 0.233* | 0.224* | 0.154** | 0.218* | 0.205* | 0.240* | 0.183* | 0.146* | 0.289* | 0.216* |
| Treatment priority 🡪 treatment adherence | 0.149* | 0.063 | 0.144* | 0.124* | 0.118* | 0.155* | 0.153* | 0.130* | 0.113* | 0.142* | 0.150* | 0.110* | 0.113** | 0.065 | 0.144* | 0.075 | 0.104** | 0.221* | 0.177* | 0.133* |
| Treatment priority 🡪 medication compliance | 0.185* | 0.220* | 0.210* | 0.181* | 0.182* | 0.208* | 0.132** | 0.202* | 0.157* | 0.200* | 0.205* | 0.174* | 0.131** | 0.133* | 0.292* | 0.211* | 0.261* | 0.177* | 0.152* | 0.191* |
| Treatment priority 🡪 treatment concerns | 0.285* | 0.257* | 0.273* | 0.258* | 0.291* | 0.223* | 0.234* | 0.269* | 0.268* | 0.264* | 0.279* | 0.257* | 0.261* | 0.141** | 0.213* | 0.199** | 0.306* | 0.366* | 0.296* | 0.263* |
| Subjective health experience (today) 🡪 health behaviour | 0.139* | 0.247* | 0.179* | 0.156* | 0.148* | 0.193* | 0.049 | 0.185* | 0.143* | 0.172* | 0.140* | 0.191* | 0.207* | 0.188* | 0.229* | 0.062 | 0.207* | 0.190* | 0.113** | 0.164* |
| Subjective health experience (today) 🡪 subjective health experience (month) | 0.430* | 0.526* | 0.544* | 0.378* | 0.454* | 0.454* | 0.489* | 0.445* | 0.551* | 0.429* | 0.400* | 0.524* | 0.516* | 0.450* | 0.499* | 0.471* | 0.418* | 0.413* | 0.478* | 0.454* |
| Subjective health experience (month) 🡪 health behaviour | 0.169* | 0.136* | 0.123* | 0.192* | 0.176* | 0.151* | 0.244* | 0.151* | 0.183* | 0.157* | 0.163* | 0.138* | 0.177* | 0.161* | 0.047 | 0.162* | 0.163* | 0.205* | 0.194* | 0.166* |
| **Indirect relationship** |  |  |  |  |  |  |  |  |  |  |  |  |  |  |  |  |  |  |  |  |
| Health consciousness 🡪 treatment adherence | 0.195* | 0.175* | 0.196* | 0.190* | 0.197* | 0.184* | 0.173* | 0.195* | 0.164* | 0.201* | 0.208* | 0.180* | 0.195* | 0.207* | 0.154* | 0.163* | 0.247* | 0.176* | 0.206* | 0.191* |
| Health consciousness 🡪 health behaviour 🡪 treatment adherence | 0.107* | 0.119* | 0.102* | 0.115* | 0.110* | 0.110* | 0.079* | 0.113* | 0.110* | 0.107* | 0.107* | 0.103* | 0.133* | 0.121* | 0.072** | 0.112* | 0.147* | 0.113* | 0.087* | 0.109* |
| Health consciousness 🡪 control 🡪 subjective health experience (today) 🡪 subjective health experience (month) 🡪 health behaviour 🡪 treatment adherence | 0.001* | 0.002** | 0.001** | 0.002* | 0.001* | 0.001* | 0.001 | 0.001* | 0.002** | 0.001* | 0.001* | 0.001** | 0.004** | 0.003** | 0.000 | 0.001 | 0.001 | 0.002** | 0.001 | 0.001* |
| Health consciousness 🡪 control 🡪 subjective health experience (month) 🡪 health behaviour 🡪 treatment adherence | 0.001** | 0.001 | 0.001 | 0.001** | 0.001** | 0.001 | 0.001 | 0.001** | 0.001 | 0.001** | 0.001** | 0.001 | 0.000 | 0.001 | 0.000 | 0.000 | 0.002 | 0.001 | 0.002 | 0.001** |
| Health consciousness 🡪 control 🡪 acceptance 🡪 subjective health experience (today) 🡪 subjective health experience (month) 🡪 health behaviour 🡪 treatment adherence | 0.001* | 0.002** | 0.001* | 0.001* | 0.002* | 0.001* | 0.002** | 0.001* | 0.002** | 0.001* | 0.001* | 0.001* | 0.003** | 0.001** | 0.000 | 0.001** | 0.002** | 0.002* | 0.001** | 0.001* |
| Health consciousness 🡪 medication compliance 🡪 treatment adherence | 0.040* | 0.011 | 0.039* | 0.031* | 0.041* | 0.025** | 0.044** | 0.032* | 0.027** | 0.036* | 0.048* | 0.034** | 0.016 | 0.049* | 0.026 | 0.013 | 0.051* | 0.011 | 0.062* | 0.034* |
| Health consciousness 🡪 control 🡪 acceptance 🡪 subjective health experience (month) 🡪 health behaviour 🡪 treatment adherence | 0.002* | 0.002** | 0.001** | 0.003* | 0.002* | 0.002** | 0.003** | 0.002* | 0.002** | 0.002* | 0.002* | 0.001** | 0.003** | 0.002** | 0.000 | 0.002** | 0.003 | 0.003* | 0.000 | 0.002* |
| Health consciousness 🡪 treatment priority 🡪 treatment adherence | 0.032* | 0.014 | 0.040* | 0.022* | 0.029* | 0.026* | 0.039** | 0.027* | 0.011 | 0.036* | 0.035* | 0.025* | 0.017** | 0.014 | 0.029** | 0.018 | 0.019** | 0.032* | 0.051* | 0.029* |
| Health consciousness 🡪 treatment priority 🡪 treatment concerns 🡪 medication compliance 🡪 treatment adherence | -0.006* | -0.002** | -0.010* | -0.003* | -0.008* | -0.003* | -0.007** | -0.006* | -0.002** | -0.007* | -0.007* | -0.006* | -0.001 | -0.003** | -0.004** | -0.005** | -0.002** | -0.003** | -0.017* | -0.006* |
| Health consciousness 🡪 control 🡪 subjective health experience (today) 🡪 health behaviour 🡪 treatment adherence | 0.002* | 0.007* | 0.002** | 0.003* | 0.002* | 0.003* | 0.001 | 0.003* | 0.002** | 0.003* | 0.002* | 0.003** | 0.009** | 0.007** | 0.002 | 0.000 | 0.003** | 0.004** | 0.001 | 0.003* |
| Health consciousness 🡪 treatment priority 🡪 medication compliance 🡪 treatment adherence | 0.016* | 0.013* | 0.023* | 0.012* | 0.018* | 0.014* | 0.013** | 0.017* | 0.006** | 0.021* | 0.020* | 0.016* | 0.007 | 0.009** | 0.024* | 0.022* | 0.015* | 0.011** | 0.019** | 0.017* |
| Health consciousness 🡪 treatment priority 🡪 treatment concerns 🡪 treatment adherence | -0.002** | -0.001 | -0.006* | -0.001 | -0.004** | -0.000 | -0.003 | -0.002** | -0.000 | -0.003** | -0.003** | -0.001 | -0.002 | -0.002 | -0.000 | -0.002 | -0.001 | -0.003 | -0.003 | -0.002** |
| Health consciousness 🡪 control 🡪 acceptance 🡪 subjective health experience (today) 🡪 health behaviour 🡪 treatment adherence | 0.002* | 0.006* | 0.003* | 0.003* | 0.003* | 0.004* | 0.001 | 0.004* | 0.003** | 0.003* | 0.002* | 0.004* | 0.006** | 0.003** | 0.004** | 0.001 | 0.006** | 0.004** | 0.002 | 0.003* |
| **Explained variance** | **31.9%** | **22.6%** | **34.0%** | **30.4%** | **32.0%** | **31.8%** | **26.6%** | **32.8%** | **27.3%** | **32.9%** | **33.1%** | **30.7%** | **30.7%** | **30.2%** | **30.4%** | **34.7%** | **29.5%** | **35.1%** | **38.5%** | **31.8%** |

*p < .001

**p < .05
